# Arabic Translation and Validation of Olfactory-Specific Quality of Life Assessment Questionnaire

**DOI:** 10.7759/cureus.16000

**Published:** 2021-06-28

**Authors:** Hoda Alsayid, Sarah Alnakhli, Hani Z Marzouki, Rickul Varshney, Faisal Zawawi

**Affiliations:** 1 Otolaryngology - Head and Neck Surgery, King Abdulaziz University, Jeddah, SAU; 2 Faculty of Medicine, King Abdulaziz University, Jeddah, SAU; 3 Otolaryngology - Head and Neck Surgery, McGill University, Montreal, CAN

**Keywords:** anosmia, arabic, quality of life, questionnaire, olfaction

## Abstract

Background: Olfaction plays a critical role in our health, emotions, social life and safety, which is why olfactory dysfunction has a great impact on a person’s life. This has been highlighted with the recent coronavirus disease 2019 (COVID-19) pandemic. Despite Arabic being the fifth most commonly spoken language and one of the six official languages of the United Nations, there is no Arabic version for an olfactory-specific quality of life assessment tool.

Method: The Questionnaire of Olfactory Disorders-Negative Statements (QOD-NS) is a validated questionnaire that assesses many aspects of a patient’s daily life. We translated this questionnaire to the Arabic language following European Organisation for Research and Treatment of Cancer (EORTC) Quality of Life Group Translation Procedure guidelines. A pilot-testing of the Arabic version was done among 20 participants, 10 of whom were confirmed to have normosmia based on scoring at least 11/12 on the Sniffin’ Sticks (SS) olfactory testing (Group 1) and another 10 participants who reported anosmia and scored less than 7/12 on the SS test. Patients could agree, partially agree, partially disagree, or disagree with each questionnaire statement.

Results: The pilot study revealed that participants with confirmed anosmia had higher questionnaire scores compared to participants with normosomia (median 22 compared to 1, p value < 0.001). For each statement on the Arabic questionnaire, all questions scored at least 80% of intra-rater reliability, and the overall intra-rater reliability was 90%.

Conclusion: The Arabic translation of QOD-NS is a validated questionnaire that can be used both in academic and clinical practice.

## Introduction

Olfaction, or sense of smell, is the oldest chemical sense [1]. Olfaction is a long, complicated process that starts with odorants dissolving in the olfactory epithelium until they finally reach the olfactory cortex, which processes these stimuli as a smell.

Olfaction plays a critical role in our lives and acts as a safety mechanism warning us of potential harmful toxins, smoke, fire, and spoiled food, all of which can be life-threatening conditions. Olfaction also has a role in social life, as it is linked to emotions and evokes memories. It is also related to personal hygiene, which can make people with olfactory dysfunction socially insecure and isolated [2]. Moreover, it has an impact on nutritional [2-3], cultural [4], spiritual [4], and psychological aspects of our lives [3, 5]. The recent coronavirus disease 2019 (COVID-19) pandemic has highlighted the impact of anosmia on our quality of life.

Olfactory dysfunction is generally divided into two broad categories: the first is quantitative, which includes anosmia (total loss of the sense of smell) and hyposmia (partial loss of smell or reduced ability). The second category is qualitative disorders, which include parosmia (distortion of the sense of smell) and phantosmia (a form of olfactory hallucination).

The most common causes of olfactory dysfunction are post-upper respiratory tract infection, sinusitis, and head trauma [6]. Eighty percent of cases are due to viral illness and chronic rhinosinusitis [7]. Other causes include tumors, substance abuse, and preclinical symptoms for some diseases, such as Parkinson’s disease and other neurological diseases.

One of the validated quality of life tools in olfactory dysfunction assessment is the Questionnaire of Olfactory Disorders-Negative Statements (QOD-NS). It has 17 negative statements that cover different aspects of patients’ daily lives [8]. To the best of our knowledge, to this date, there has been no validated Arabic version of a questionnaire for the evaluation of the olfactory-specific quality of life. This is important as chronic rhinosinusitis is very common in Arabic-speaking countries. The purpose of this study is to translate the questionnaire into Arabic. This validated questionnaire was adapted to be used in both clinical practice and research in Arabic-speaking patients.

## Materials and methods

This study received institutional ethics approval. The QOD-NS consists of 17 statements, and each statement has a scale containing four statements: I agree, I agree partially, I disagree partially, and I disagree. The answers were assigned a score of 3, 2, 1, and 0, respectively. Approval of translation and usage was acquired from the Department of Otorhinolaryngology, Smell and Taste Clinic, University of Dresden Medical School, Fetscherstr in Germany. The translation was performed following the European Organisation for Research and Treatment of Cancer (EORTC) Quality of Life Group Translation Procedure, fourth edition guidelines [9].

First, the translation was performed from English to the Arabic language by two native Arabic speakers who worked independently. Then, it was reviewed by a third Arabic native speaker. The third Arabic native speaker combined the two translations or chose the best between the two, to come up with the Arabic questionnaire. The final questionnaire was approved by the three individuals.

**Figure 1:**
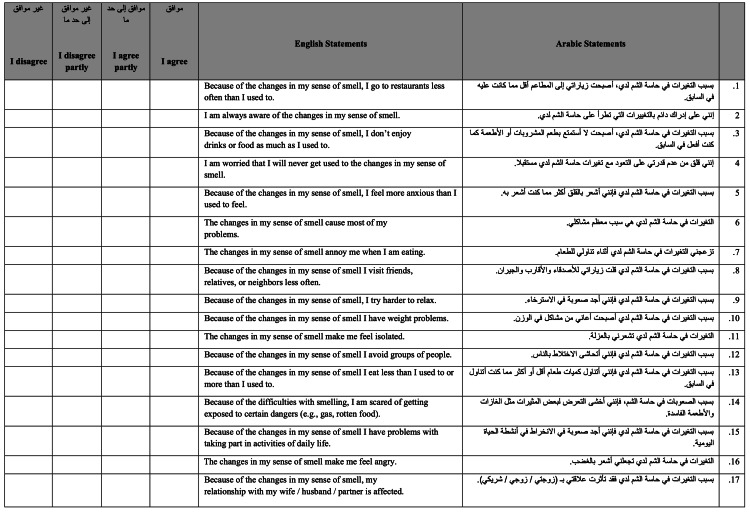
Version 1. This is the first version of the questionnaire after translation. Scoring system: I disagree = 0, I disagree partly = 1, I agree partly = 2, I agree = 3 NOTE: The version that was sent to participants did not include the English translation. This was added for publication and readers' understanding

After this stage, the Arabic questionnaire (version 1) was distributed to 15 individuals (Figure 1). One of our team members was assigned to meet with each participant to get feedback regarding difficulties with any of the statements. 

**Figure 2:**
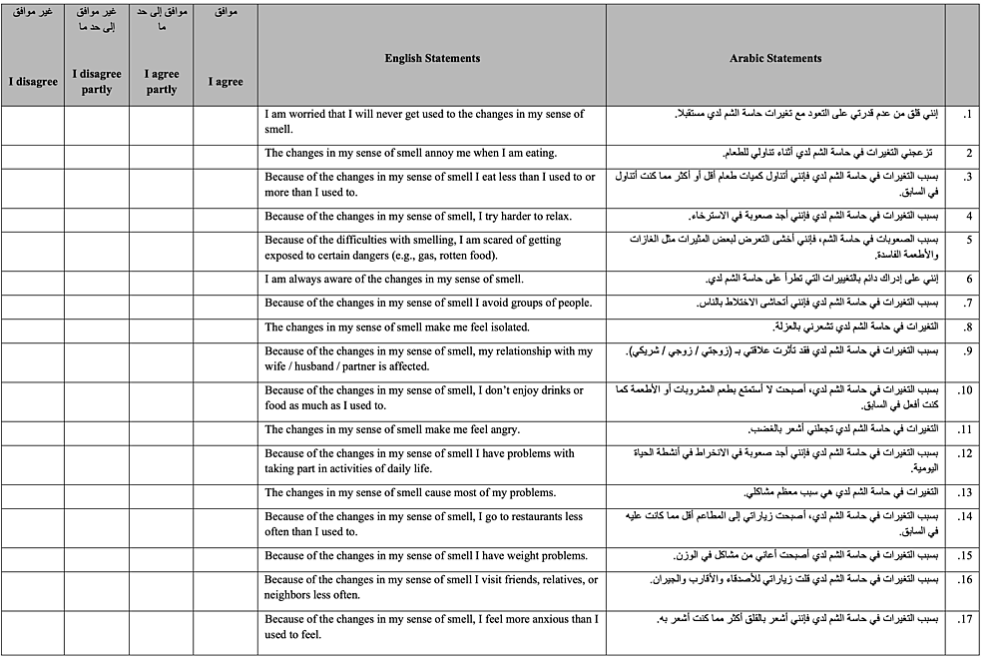
Version 2. This is the second version of the questionnaire. Same questions but rearranged in different order. The second version was given to the same group of participants two weeks after the first version was given. Scoring system: I disagree = 0, I disagree partly = 1, I agree partly = 2, I agree = 3 NOTE: The version that was sent to participants did not include the English translation. This was added for publication and readers' understanding

A step was added in our study that was not in the EORTC guidelines. In this step, which was performed after two weeks, the same Arabic questionnaire draft but rearranged in a different order was distributed among the same 15 individuals (Figure 2). They did not receive help from any team member. This was done to ensure that they did not have any difficulties in answering the questionnaire by themselves. A comparison was then made between versions 1 and 2 to determine if the answers were the same in most participants. Finally, a backward translation was performed from Arabic to the English language by a certified service. After reviewing all the processes, a final Arabic version of the QOD-NS was established.

**Figure 3:**
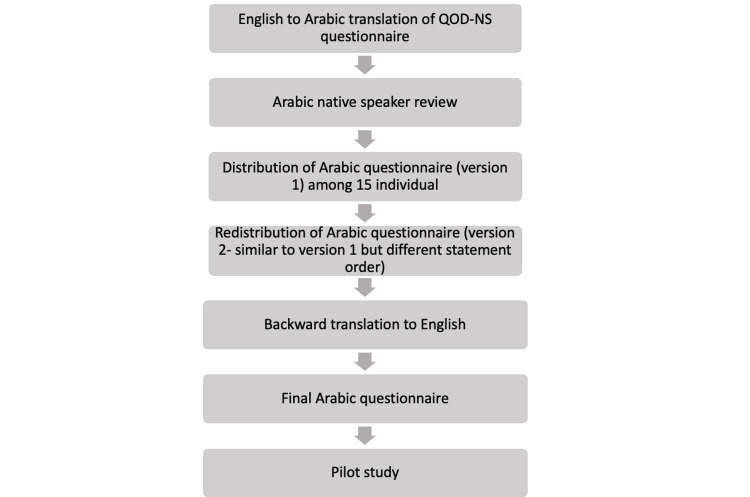
Methodology flowchart. This is the flowchart of the methodology steps that were performed to translate and validate the survey.

The final step was a pilot study. This was performed by recruiting 20 adult participants (18 years and older), 10 people had anosmia and 10 did not (Figure 1). The grouping was decided based on the patient-reported sense of olfaction and scoring using the Sniffin’ Sticks (SS) Universal Olfaction Test. For a participant to be in Group 1 (normosmia) they had to report normal olfaction and a score of at least 11/12 on the SS. Group 2 participants had to report a reduced sense of smell as well as a score of 6/12 or less on the SS. Statistical analysis was performed using SPSS© 26 (IBM, Armonk, NY). Mann-Whitney U test was utilized to determine the significance (Figure 3). 

## Results

The Arabic questionnaire was distributed among 15 individuals who were between 20 and 60 years old. Eight of the participants were female, and seven were male. Feedback was taken from each participant, and no difficulty was recognized with any of the 17 Arabic statements.

After two weeks, the same Arabic statements but in different orders were distributed among the same group. After comparing the answers of each participant on both versions, based on the scale ranging containing I agree, I agree partially, I disagree partially, and I disagree for each statement, all questions scored at least 80% intra-rater reliability. The overall intra-rater reliability was 90%. (Table 1).

**Table 1:** Intra-rater reliability. This table highlights the intra-rater reliability of each of the questions and the overall intra-rater reliability. None of the questions had an intra-rater reliability less than 80% and the overall intra-rater reliability was 90%.

Arabic statements	Two-weeks intra-rater reliability
بسبب التغيرات في حاسة الشم لدي، أصبحت زياراتي إلى المطاعم أقل مما كانت عليه في السابق.	100%
إنني على إدراك دائم بالتغييرات التي تطرأ على حاسة الشم لدي.	80%
بسبب التغيرات في حاسة الشم لدي، أصبحت لا أستمتع بطعم المشروبات أو الأطعمة كما كنت أفعل في السابق.	80%
إنني قلق من عدم قدرتي على التعود مع تغيرات حاسة الشم لدي مستقبلا.	80%
بسبب التغيرات في حاسة الشم لدي فإنني أشعر بالقلق أكثر مما كنت أشعر به.	86%
التغيرات في حاسة الشم لدي هي سبب معظم مشاكلي.	93%
تزعجني التغيرات في حاسة الشم لدي أثناء تناولي للطعام.	80%
بسبب التغيرات في حاسة الشم لدي قلت زياراتي للأصدقاء والأقارب والجيران.	93%
بسبب التغيرات في حاسة الشم لدي فإنني أجد صعوبة في الاسترخاء.	86%
بسبب التغيرات في حاسة الشم لدي أصبحت أعاني من مشاكل في الوزن.	93%
التغيرات في حاسة الشم لدي تشعرني بالعزلة.	100%
بسبب التغيرات في حاسة الشم لدي فإنني أتحاشى الاختلاط بالناس.	100%
بسبب التغيرات في حاسة الشم لدي فإنني أتناول كميات طعام أقل أو أكثر مما كنت أتناول في السابق.	100%
بسبب الصعوبات في حاسة الشم، فإنني أخشى التعرض لبعض المثيرات مثل الغازات والأطعمة الفاسدة.	93%
بسبب التغيرات في حاسة الشم لدي فإنني أجد صعوبة في الانخراط في أنشطة الحياة اليومية.	93%
التغيرات في حاسة الشم لدي تجعلني أشعر بالغضب.	93%
بسبب التغيرات في حاسة الشم لدي فقد تأثرت علاقتي بـ (زوجتي / زوجي / شريكي).	86%
Overall intra-rater reliability	90%

On the pilot study results, the median age of participants was 25 years (range 19-40 years). Group 2 (anosmia) had significantly higher test questionnaire scores with a median score of 22 compared to participants in Group 1 (normosmia) with a median score of 1 (p value < 0.001).

## Discussion

Olfaction is one of the special senses that plays a major role in our health, emotions, social life, and safety. That is why olfactory dysfunction has a great impact on a person’s life. As olfaction is closely related to the quality of life, some studies have shown a connection between olfaction and depression [10-11].

In a study performed on 42,000 participants in 1998 by the National Health Interview Survey in the United States, 1.4% of participants reported olfactory dysfunction [12]. Additionally, 4.9% of the 7,306 participants in the Korean National Health Survey in 2013 reported olfactory dysfunction [13], and 19.1% of participants from the Swedish population reported dysfunction [3]. The prevalence of olfactory dysfunction in different age groups was estimated in different studies. The prevalence was between 40% and 70% among elderly individuals [14-15] and 5%-15% in the younger age group [3, 16]. Unfortunately, no data are available on the prevalence of olfactory dysfunction in any Arabic-speaking country.

One of the major causes of olfactory dysfunction is chronic sinusitis, and it is one of the cardinal features in the diagnosis of chronic sinusitis. Globally, the prevalence of chronic sinusitis ranges between 12% and 15%, which makes it the most common chronic inflammatory disease in the world [17-18]. In the Western world, chronic sinusitis affects 10% of the population [19]. Due to insufficient data, little is known regarding the prevalence of this disorder in the Middle East area. However, an increase in prevalence was noticed in one study in Saudi Arabia [17]. Furthermore, 40%-80% of chronic sinusitis patients were estimated to have olfactory dysfunction in one study [20].

For the assessment of olfactory function, many tests have been developed. A few of the most common tests are the University of Pennsylvania Smell Identification Test (UPSIT), SS Test, San Diego Odor Identification Test (SDOIT), Scandinavian Odor-Identification Test (SOIT), and others. All of these tests contain a specific number of odors to test the patient’s ability for odor identification, threshold, and discrimination.

In the past, most of the studies focused on disease prevalence, risk factors, mortality, and economy. Limited studies have focused on the impact of the disease on the patient’s quality of life. However, in the past two decades, an increasing number of measures and tests have become available [21]. One of the validated tools in assessing the olfactory-specific quality of life is the QOD-NS, which measures the effect of olfaction on daily life with 17 negative statements [22-24]. This validated questionnaire is a shortened version of the original olfactory-specific questionnaire of olfactory disorders developed by Frasnelli and Hummel in 2005, which had 52 items [24].

The Arabic language is one of the six official languages of the United Nations. Despite Arabic being the fifth most commonly spoken language, and the official language in 25 countries with more than 300 million Arabic speakers worldwide (reaching 422 million in some sources) [25], there is no Arabic version of an olfactory-specific quality of life assessment.

This study was done to translate a validated questionnaire from English to the Arabic language. It modified both the 17-statement questionnaire. This will lead to a better appreciation of the impact of smell disorders for patients, and therefore, better management in both clinical practice and research.

## Conclusions

Olfactory dysfunction greatly impacts patients’ quality of life. Despite Arabic being one of the most commonly spoken languages, an Arabic olfactory quality of life assessment tool has still not been available. Adapting one of the validated questionnaires and translating it to Arabic was the goal of this article. The outcomes of the current study support the use of the Arabic translation of the QOD-NS in both academic and clinical practice.
